# Nano-pulling stimulates axon regeneration in dorsal root ganglia by inducing stabilization of axonal microtubules and activation of local translation

**DOI:** 10.3389/fnmol.2024.1340958

**Published:** 2024-04-03

**Authors:** Alessandro Falconieri, Pietro Folino, Lorenzo Da Palmata, Vittoria Raffa

**Affiliations:** Department of Biology, Università di Pisa, Pisa, Italy

**Keywords:** nerve regeneration, magnetic nanoparticles, dorsal root ganglia, microtubules, local translation

## Abstract

**Introduction:**

Axonal plasticity is strongly related to neuronal development as well as regeneration. It was recently demonstrated that active mechanical tension, intended as an extrinsic factor, is a valid contribution to the modulation of axonal plasticity.

**Methods:**

In previous publications, our team validated a the “nano-pulling” method used to apply mechanical forces to developing axons of isolated primary neurons using magnetic nanoparticles (MNP) actuated by static magnetic fields. This method was found to promote axon growth and synaptic maturation. Here, we explore the use of nano-pulling as an extrinsic factor to promote axon regeneration in a neuronal tissue explant.

**Results:**

Whole dorsal root ganglia (DRG) were thus dissected from a mouse spinal cord, incubated with MNPs, and then stretched. We found that particles were able to penetrate the ganglion and thus become localised both in the somas and in sprouting axons. Our results highlight that nano-pulling doubles the regeneration rate, and this is accompanied by an increase in the arborizing capacity of axons, an accumulation of cellular organelles related to mass addition (endoplasmic reticulum and mitochondria) and pre-synaptic proteins with respect to spontaneous regeneration. In line with the previous results on isolated hippocampal neurons, we observed that this process is coupled to an increase in the density of stable microtubules and activation of local translation.

**Discussion:**

Our data demonstrate that nano-pulling enhances axon regeneration in whole spinal ganglia exposed to MNPs and external magnetic fields. These preliminary data represent an encouraging starting point for proposing nano-pulling as a biophysical tool for the design of novel therapies based on the use of force as an extrinsic factor for promoting nerve regeneration.

## 1 Introduction

Nerve injuries are a serious cause of disability throughout the world and have a considerable socio-cultural and economic impact. Contusion-related injuries such as falls, road and industrial accidents are some of the most common causes, but other injuries also include cuts (e.g., knives, saw blades, fans, glass) or bone fractures (Robinson, 2000; Burnett and Zager, 2004; Campbell, 2008). Nerve compression syndromes, i.e., structural and functional alterations in the nerve or adjacent tissue due to load or pressure, are other typical causes of nerve injuries (Rempel et al., 1999; Burnett and Zager, 2004; Hochman and Zilberfarb, 2004; Campbell, 2008).

There is currently no universal method for treating nerve injuries. Unfortunately, there are some hard-to-treat injuries such brain and spinal cord injuries, and most of these diseases are considered incurable. The use of nanotechnology in nerve regeneration therapy has gained increasing interest. The main uses include the production of biomaterials (natural or synthetic) for the development of scaffolds and nerve guidance conduits (Gerth et al., 2015; Sarker et al., 2018), or for implementing smart drug delivery (Tajdaran et al., 2019; Bianchini et al., 2023). Of these, MNPs have been effectively used for the delivery of growth factors, such as nerve growth factor (NGF) (Ziv-Polat et al., 2014; Zuidema et al., 2015; Giannaccini et al., 2017; Marcus et al., 2018), brain-derived neurotrophic factor (BDNF) (Pilakka-Kanthikeel et al., 2013; Wise et al., 2016), glial cell line-derived neurotrophic factor (GDNF) (Ziv-Polat et al., 2014), vascular endothelial growth factor (VEGF) (Giannaccini et al., 2017) to promote nerve survival and neurite regeneration (Zuidema et al., 2015; Wise et al., 2016; Giannaccini et al., 2017).

Our team recently proposed a new application of MNPs for the mechanical stimulation of neurons (De Vincentiis et al., 2020). Historically, chemical signaling was thought to be mainly responsible for neuronal growth and differentiation. However, the role of mechanical force in guiding and promoting these same mechanisms has been now recognized (Suter and Miller, 2011; Franze, 2013; Franze et al., 2013). MNPs have the advantage of exerting extremely low forces, similarly to endogenous ones (picoNewton order).

Since the early 21st century, many groups have exploited MNPs to modulate axonal functions, such as axon specification and orientation (Riggio et al., 2014; Kunze et al., 2015; Raffa et al., 2018), intracellular calcium dynamics (Tay et al., 2016; Tay and Di Carlo, 2017; De Vincentiis et al., 2020), cytoskeletal dynamics (Pita-Thomas et al., 2015; De Vincentiis et al., 2020; Falconieri et al., 2023b) axonal transport (Steketee et al., 2011; Chowdary et al., 2013; Kunze et al., 2017; Chowdary et al., 2019; Falconieri et al., 2023b), elongation and branching (Steketee et al., 2011; Riggio et al., 2014; Tay et al., 2016; Raffa et al., 2018; De Vincentiis et al., 2020; Wang et al., 2020; Falconieri et al., 2023b), and neuron maturation (De Vincentiis et al., 2020, 2023; Falconieri et al., 2023b).

In our previous studies performed on isolated hippocampal neurons, we found that nano-pulling induces a remodelling of the axonal cytoskeleton, by increasing the number of microtubules (MTs) and the fraction of stable MTs (De Vincentiis et al., 2020; Falconieri et al., 2023b). We suggested that the structural changes at the level of MTs modulate axonal transport and activate local translation, stimulating axon outgrowth and synaptic maturation (Falconieri et al., 2023a, 2023b).

These interesting results raise the question as to whether nano-pulling is important for translational research, in terms of its possible use for studying signal mechanotransduction. In fact, since many MNP-based nano-formulations and magnetic fields have been approved for clinical use, nano-pulling could be used as a non-invasive medical tool/device. However, it is still not clear: (i) how MNPs interact *in vivo*, (ii) whether they are able to penetrate neuronal tissues, (iii) whether they can reach the regenerating axons, and (iv) how they promote their regeneration. This lack of knowledge hinders the effective translation of the technology in pre-clinical models.

Here, we propose a study on DRG, which are bilateral structures located between peripheral nerve terminals and the dorsal horn of the spinal cord, that carry sensory information from the periphery (peripheral nervous system, PNS) to the central nervous system (CNS) (Berta et al., 2017; Ahimsadasan et al., 2018). Specifically, in each ganglion there are neurons involved in non-noxious sensation, including light touch, vibration and proprioception while others are involved in noxious sensation (i.e., nociceptors), for the detection of stimuli associated with pain, but also with thermal and mechanical responses (Mantyh, 2006; Berta et al., 2017). DRG contain not only neurons but also non-neuronal cells (glial cells, endothelial cells, fibroblasts), recapitulating all the complexity of a nerve tissue (Haberberger et al., 2019). Specifically, DRG are made up of a specific form of glia, called satellite cells, which create an envelope around cell bodies of sensory neurons that project fibers, surrounded by connective tissue and blood vessels (Haberberger et al., 2019). DRG neurons are a pseudo-unipolar type of sensory neurons with two branches (the distal and proximal process), one projecting into the CNS and the other into the PNS. DRG thus represent an ideal model system for a pilot study of nerve regeneration, since the dissection maintains the intact structure of the ganglion, but the distal process and the proximal processes are resected and their regeneration can be studied under controlled conditions (Nascimento et al., 2018).

In the present study, this model was used to evaluate the effects of mechanical stimulation mediated by externally-administered MNPs, regardless of the anatomical compartments (CNS *vs* PNS) which are known to present a different predisposition to regeneration (Goldberg and Barres, 2000; Huebner and Strittmatter, 2009; Mietto et al., 2015).

## 2 Methods

### 2.1 Animals

All the animal procedures were performed in compliance with protocols approved by Italian Ministry of Public Health and of the local Ethical Committee of University of Pisa, in conformity with the Directive 2010/63/EU. Post-natal day (P) 3 C57BL/6 J were used. Both male and female mice were purchased from Charles River Laboratories [Charles River Laboratories, Italy, Calco (LC)]. They were maintained in a controlled environment (23 ± 1°C, 50 ± 5% humidity) with a 12 / 12 h light / dark cycle with food and water *ad libitum*.

### 2.2 Magnetic nanoparticles

For magnetic stimulation of DRG, MNPs were used (Fluid-MAG-ARA, Chemicell, Germany). As stated from the supplier, MNPs were characterized by a core of iron oxide approximately 75 ± 10 nm in diameter, saturation magnetization of 59 Am^2^/kg^−1^ and a hydrodynamic diameter of 100 nm. The outer layer is made of glucuronic acid and represents an organic shell to avoid nanoparticles aggregation.

### 2.3 Dorsal root ganglia organotypic cultures

For DRG organotypic cultures P3 mice were used. For dissection, isolation and culturing we modified a protocol proposed by Han and colleagues (Han et al., 2020). Briefly, animals were sacrificed and their columns were excised in a dissection medium constituted of a solution of D-glucose 6.5 mg ml^−1^ in DPBS (Gibco; Thermo Fisher Scientific, Waltham, Massachusetts, US; #14190–144). The spinal cords were removed and DRG from cervical and thoracic regions were collected. Then, the DRG have been stripped of their nerve roots which branch off from the body and placed on glass coverslips previously coated with 500 μg ml^−1^ poly-L-lysine (PLL, Sigma-Aldrich, Burlington, Massachusetts, US, #P4707) and 10 μg ml^−1^ laminin (Gibco, Thermo Fisher Scientific, Waltham, Massachusetts, US, #23017–015). To promote adhesion, the ganglia were placed on ice for 45 min. Then, culture medium consisting of Neurobasal-A medium (Gibco, Thermo Fisher Scientific, Waltham, Massachusetts, US, #12349–015) modified with B27 (Gibco, Thermo Fisher Scientific, Waltham, Massachusetts, US, #17504–044), 2 mM Glutamax (Gibco, Thermo Fisher Scientific, Waltham, Massachusetts, US, #35050–038), 50 IU·ml^−1^ penicillin, 50 μg·ml^−1^ streptomycin was added. After 4 h, fresh cell culture medium supplemented with 5 μg ml^−1^ MNPs was added, and the samples were incubated at 37°C in a humidity-saturated atmosphere containing 95% air and 5% CO2.

### 2.4 Magnetic stimulation

Magnetic stimulation was applied by a Halbach-like cylinder magnetic applicator that provides a constant magnetic field gradient (46.5 T m^−1^) in the radial centrifugal direction (Riggio et al., 2014; Raffa et al., 2018). All the experiments were carried out by placing 35 mm Petri dishes, containing the glass coverslips, inside of the applicator to develop a constant, static and permanent force on the DRG. Mechanical forces, magnetically-actuated, were applied from Day *in vitro* (DIV) 1 to DIV3.

### 2.5 Nano-pulling

DRG (four / five per glass coverslip) were placed in 35 mm Petri dishes at DIV0. After 4 h from the complete attachment, MNPs were added (DIV0.17). All the DRG considered in this study received MNPs, but only the stretched groups were exposed to the magnetic field. At DIV1, samples placed inside of the magnetic applicator (stretched groups; Stretch) or outside (control groups; Ctrl). After 48 h of incubation (DIV3), all the samples were fixed and prepared for fluorescence microscopy.

### 2.6 Ribopuromycylation

We evaluated the population of ribosomes, actively translating, in DRG by the ribopuromycylation (RPM) method, modifying a protocol already published (Falconieri et al., 2023b) [in turn, modified from (Bastide et al., 2018)]. Briefly, DRG were harvested and seeded on glass coverslips at DIV0. After MNP delivery (DIV0.17) and nano-pulling (from DIV1 to DIV3), the ganglia were treated with 200 μM emetine (Sigma-Aldrich, Burlington, Massachusetts, US, #E2375) and 100 μM puromycin (Sigma-Aldrich, Burlington, Massachusetts, US, #P8833) for 10 min at 37°. Then, ganglia were washed with ice-cold 0.0003% digitonin (Sigma-Aldrich, Burlington, Massachusetts, US; #D141) in DPBS for 2 min. Next, samples were first washed with ice-cold DPBS and then fixed in 4% PFA, 4% sucrose (Sigma-Aldrich, Burlington, Massachusetts, US, #S0389) for 30 min at RT.

### 2.7 Immunostaining

At DIV3, DRG were fixed in 4% PFA and 4% sucrose (Sigma-Aldrich, Burlington, Massachusetts, US, #S0389) for 30 min at room temperature (RT). For studying axon regeneration, samples were permeabilized with 0.5% Triton X-100 in DPBS for 20 min and blocked in 5% GS / 0.3% Triton X-100 in DPBS for 45 min. BTUBBIII antibody (Sigma-Aldrich, Burlington, Massachusetts, US, #T8578, 1:500) was diluted in 3% GS / 0.2% Triton X-100 in PBS overnight (ON). The day after, samples were washed and incubated with secondary antibody (Thermo Fisher Scientific, Waltham, Massachusetts, US, #06380 or #AB_2633280, 1:500) and Hoechst 33342 (Thermo Fisher Scientific, Waltham, Massachusetts, US, #H3570, 1:1000) for 1 h at RT.

For staining organelles and acetylated / tyrosinated MTs, we followed a protocol already published (Cioni et al., 2019). Briefly, after fixation, ganglia were removed to study organelle dynamics in the axonal component. Then, samples were permeabilized in 0.1% Triton X-100 in PBS for 5 min. Blocking was performed by incubate samples in 5% GS in DPBS for 30 min at RT. Primary antibodies (BTUBBIII, Sigma-Aldrich, Burlington, Massachusetts, US, #T8578, 1:500; BTUBBIII, Abcam, Cambridge, UK, #ab41489, 1:1000; acetylated tubulin, Sigma-Aldrich, Massachusetts, US, #T7451, 1:400; tyrosinated tubulin, Abcam, Cambridge, UK, # ab6160, 1:400; KDEL, Thermo Fisher Scientific, Waltham, Massachusetts, US, #PA1-013, 1:200; TOMM20, Abcam, Cambridge, UK, #ab86735, 1:200; TOMM20, Abcam, Cambridge, UK, #ab289670, 1:200; Puromycin, Sigma-Aldrich, Burlington, Massachusetts, US, #MABE343, 1:1000; S6, Cell Signaling, Danvers, Massachusetts, US, #22175, 1:200; Synapsin I, Synaptic Systems, Goettingen, Germany, #106103, 1:500) were diluted in DPBS overnight (ON) at 4°C. The day after, samples were washed and incubated with secondary antibodies (Thermo Fisher Scientific, Waltham, Massachusetts, US, #A11029, #R6393, #A21236, #A11006, #A11008, #A11011, #A21244, #A11041, #A21449, 1:500) and Hoechst 33342 (Thermo Fisher Scientific, Waltham, Massachusetts, US, H1399, 1:1000). Images were acquired using a fluorescent microscope (Nikon, TE2000-U) for nano-pulling assays or a laser scanning confocal microscope (Nikon, Eclipse Ti) for mitochondria, endoplasmic reticulum (ER), synaptic marker, and ribosomes quantification.

### 2.8 Samples preparation for transmission electron microscopy

Ultrastructural characterization was carried out in transmission electron microscopy (TEM) following a protocol already published (De Vincentiis et al., 2020). Briefly, DRG were fixed in 1.5% glutaraldehyde in 0.1 M sodium cacodylate buffer (pH 7.4). After fixation, the ganglia were detached from glass coverslips and the different compartments (ganglion / axons) were processed separately. Then, samples were postfixed in reduced osmium solution (1% OsO4, 1% K3Fe(CN)6, and 0.1 M sodium cacodylate buffer). Staining was performed with our homemade solution (Moscardini et al., 2020). Samples were dehydrated in a growing series of ethanol, and flat-embedded in Epoxy resin (Epoxy embedding medium kit, Merck KGaA, Darmstadt, Germany; #). UC7 LEICA ultramicrotome (UC7, Leica Microsystems) was used to cut ultra-thin sections (90 nm) that were then collected on 300 mesh copper grids (Electron Microscope Science). TEM micrographs were acquired with a TEM microscope JEM-1010 (Jeol, Tokyo, Japan) operating at 80 Kv equipped with MegaView III high-resolution digital camera with an AnalySIS imaging software (Soft Imaging System, Muenster, Germany).

### 2.9 Image analysis

To establish the different patterns of ramification of DRG organotypic cultures, the ImageJ software was used (Schneider et al., 2012). Specifically, the branching patterns were evaluated using “neuroanatomy,” a fiji plugin, through the “sholl” function (Ferreira et al., 2014). Sholl function was exploited to determine the complexity of ramifications by evaluating the number of intersections of the neurites that arise from the DRG. Briefly, DRG were binarized (“threshold” function) and using the “Sholl” function a series of concentric rings from the center of the ganglion were generated. The number of intersections of neurites in each ring were counted. For each DRG was evaluated the average of intersections of the neurites with the generated rings. The distance between subsequent rings was set on 5 μm. DRG axonal ramification was analysed from 4x magnification images. Further, we calculate the elongation rate of the control group *er_k_* and of the stretched group *er_s_* as follow:

$$er_{k}=\frac{A\left(t\right)}{t}\text{,}$$

$$er_{s}=\frac{A\left(t\right)-er_{k}.t_{0}}{t-t_{0}}$$

being *A* the axon area, *t* the time in culture, t_0_ the time when the magnet was added.

For fluorescence quantification in organelle and MT studies, we evaluated the mean fluorescence ( $\bar{f}$ ), following a method already published (Falconieri et al., 2023b). Briefly, the area (A, ROI region of interest), integrated density (IntDen, sum of all the pixel intensities in that selected region) and the mean fluorescence of background readings ( ${\bar{f}}_{\mathrm{back}}$ ) were evaluated in fiji. Specifically, we followed the formula reported here:

$$\bar{f}=\frac{\mathrm{IntDen}-\left({\bar{f}}_{\mathrm{back}}.A\right)}{A}$$

In this way, we were able to evaluate the mean fluorescence within the region of interest, i.e., the area of the ROI corresponding to the axonal compartment of the DRG. For these studies, the mean fluorescence was measured only after exclusion of the ganglion to focus only on the axonal component. Fluorescence quantification was evaluated from large 10x (ER membranes, mitochondria, ribosomes, synaptic vesicles) or 60x (MTs) magnification images. For acquisition, only axons in the semi-plane with the same orientation of force vector were considered.

### 2.10 Statistical analysis

Data were plotted with GraphPad software, version 7.0.0. Values are reported as the mean ± standard error of the mean (SEM). Outliers were eventually identified and removed using the ROUT method (Q = 1%). The normality of data distribution was tested using the Kolmogorov–Smirnov normality test. We used the *t* test for unpaired data followed by the Bonferroni correction. Mann–Whitney test was performed for non-normally distributed data. Significance was set at *p* ≤ 0.05.

## 3 Results

### 3.1 Nano-pulling promotes the addition of new mass in regenerating axons of DRG explants

**Figure 1:**
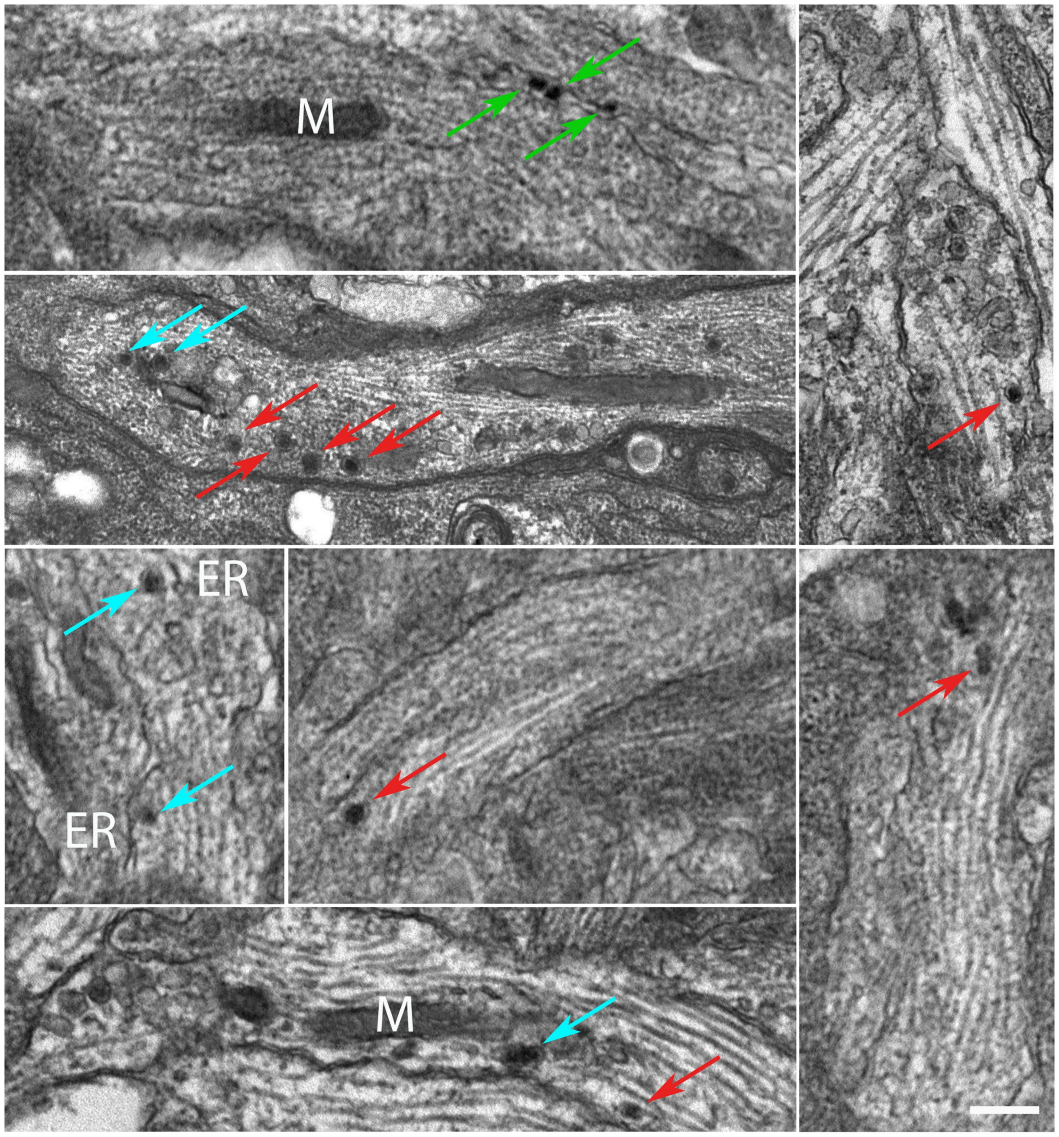
MNPs are localized in the axons of DIV3 DRG neurons. Red arrows highlight MNPs freely dispersed within the axoplasm. Green arrows show MNPs in proximity of the axonal membrane. Cyan arrows points MNPs in proximity of the sub-cellular components, e.g., endoplasmic reticulum “ER” membranes and mitochondria “M,” respectively. Scale bar: 300 nm.

DRG were explanted and the whole ganglia were put in culture (DIV0) and incubated with a MNP-modified medium after a few hours (DIV0.17). We assessed the localization of MNPs by TEM. TEM micrographs reveal that MNPs can be internalized in the ganglion (Supplementary Figure S1). In soma, MNPs were not found within membranous structures such as endosomes, lysosomes or other vesicles (Supplementary Figure S2); we never detected their presence within nuclei; they appear freely dispersed in the cytoplasm, mainly as monodispersed nanoparticles (Supplementary Figure S2, arrows). Cell membrane invaginations in proximity of MNPs, or intracellular vesicles surrounding the MNPs, were not detected. Next, we focused our analysis at the axonal level. At high magnification, MNPs appear as spherical particles with an inorganic core (iron oxide) and an organic corona (glucuronic acid) that serves to prevent aggregation. In fact, as can be seen in Figure 1, the MNPs are mainly present as single dots intracellularly. In addition, TEM micrographs show that MNPs are within the axoplasm (Figure 1; red arrows), in proximity of the axonal membrane (green arrows) or subcellular compartments such as the endoplasmic reticulum (ER) and mitochondria (m) (cyan arrows).

**Figure 2:**
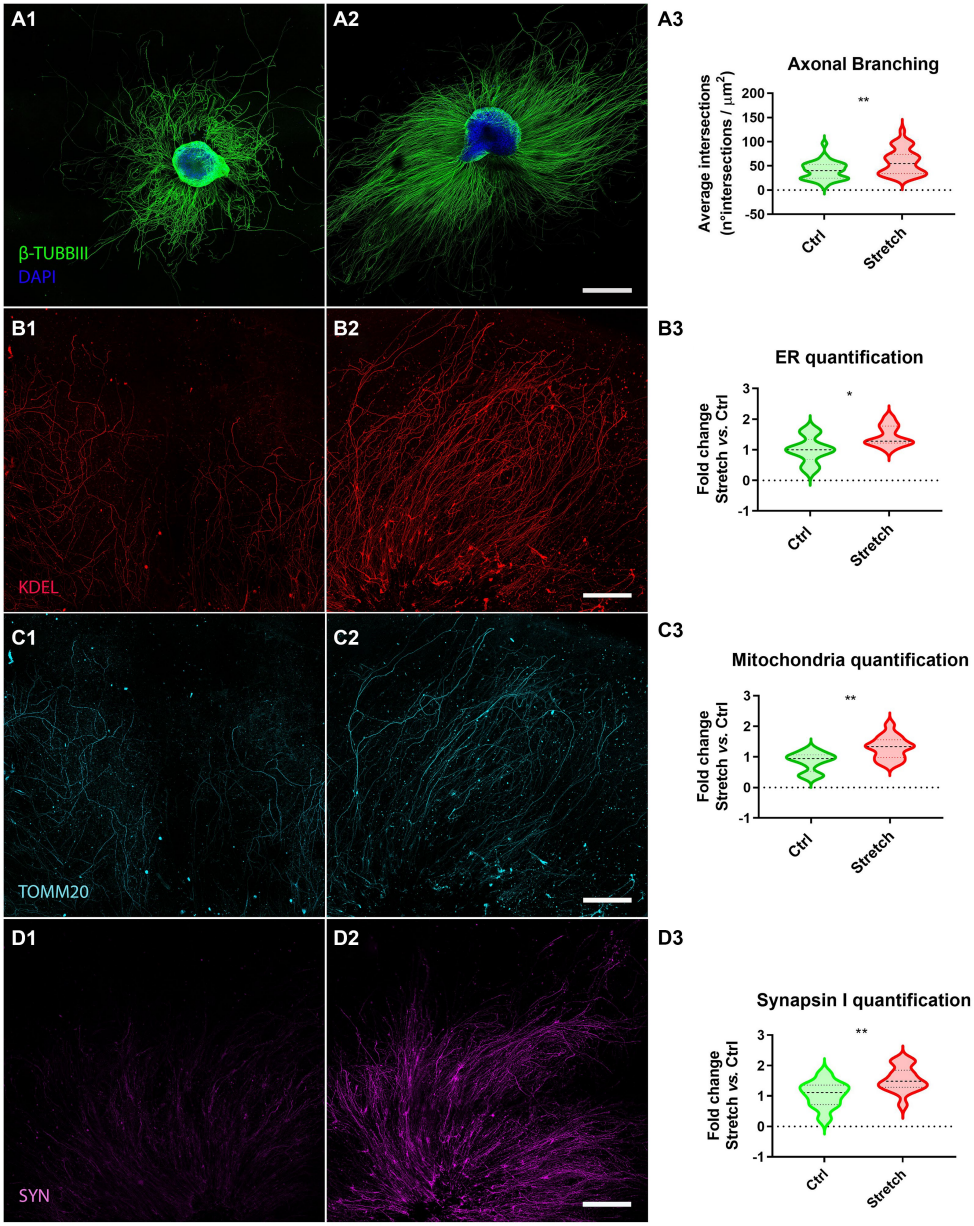
Nano-pulling promotes mass addition in DRG axons. **(A)** DIV3 DRG in unstretched **(A1)** and stretched **(A2)** conditions. Β-TUBBIII (green) and DAPI (blue) staining. **(A3)** Sholl analysis of stimulated and unstimulated DRG. Violin plot (median and extremes as 1st and 3rd quartiles). *t* test for unpaired data. *p* = 0.001. *N* > 34 DRG from 18 mice. **(B)** Immunostaining of endoplasmic reticulum membranes in unstretched **(B1)** and stretched **(B2)** DRG. KDEL (red) staining. **(B3)** Quantification of ER membranes. Violin plot (median and extremes as 1st and 3rd quartiles). *t* test for unpaired data. *p* = 0.01. *N* > 9 DRG from 6 mice. **(C)** Immunostaining of mitochondria in control **(C1)** and stimulated **(C2)** DRG. TOMM20 (cyan) staining. **(C3)** Quantification of mitochondria. Violin plot (median and extremes as 1st and 3rd quartiles). *t* test for unpaired data. *p* = 0.0016. *N* > 12 DRG from 10 mice. Outliers were identified and removed. **(D)** Immunostaining of synapsin I vesicles in control **(D1)** and stretched **(D2)** DRG. SYN (magenta) staining. **(D3)** Quantification of synapsin I vesicles. Violin plot (median and extremes as 1st and 3rd quartiles). *t* test for unpaired data. *p* = 0.0099. *N* > 10 DRG from 8 mice. Scale bars: **A** = 500 μm; **B**, **C**, **D** = 250 μm.

We randomly allocated DRG explants to two groups, the control (Ctrl; Figure 2A1) and the stretched (Stretch; Figure 2A2) groups at DIV1. The stimulation time was set at 48 h, following a procedure that we had already tested in pilot studies on isolated primary neurons and neuron-like cell cultures (Raffa et al., 2018; De Vincentiis et al., 2020). Sholl’s method was used to verify the effect of mechanical forces on the regenerating axons of DRG explants, by estimating axon branching and ramification. Specifically, we found that the nano-pulling led to a significant increase in the area covered by the axons subjected to stimulation with respect to the controls (Figure 2A3; *p* = 0.001). To further explore the axonal growth in response to tension, we evaluated the elongation rate. Specifically, we found that stretched samples have an elongation rate of 0.025 ± 0.003 μm^2^/h (*n* = 34 DRG axonal area), i.e., a 2-fold increase compared to spontaneous elongation (0.012 ± 0.001 μm^2^/h; *n* = 37 DRG axonal area; Mann–Whitney test; *p =* 0.003).

Considering that axon growth is usually accompanied by an accumulation of organelles that play a key role in lipid and protein synthesis and energy supply, we assessed the amount of ER membranes (Figure 2B) and mitochondria (Figure 2C) in the regenerating axons sprouting from the ganglion. The whole ganglion was excluded to restrict the analysis to the regenerating component. Experimental data demonstrated that axons subjected to mechanical stimulation (Figures 2B,C2) presented a statistically different number of ER membranes (Figure 2B3; *p* = 0.01) and mitochondria (2C3; *p* = 0.0016) with respect to axons in spontaneous regeneration.

We were wondering if the increase in the regeneration rate is also associated to axon maturation. Specifically, we estimated the concentration of synapsin I, as an early marker for synapse formation (Fornasiero et al., 2010), by detecting its mean fluorescence in the regenerating DRG axons. We found that the nano-pulling induces an increase in synapsin I signal, compared to the control condition of spontaneous regeneration (Figure 2D3; *p* = 0.0099).

### 3.2 Nano-pulling promotes the activation of local translation in regenerating axons of DRG explants

**Figure 3:**
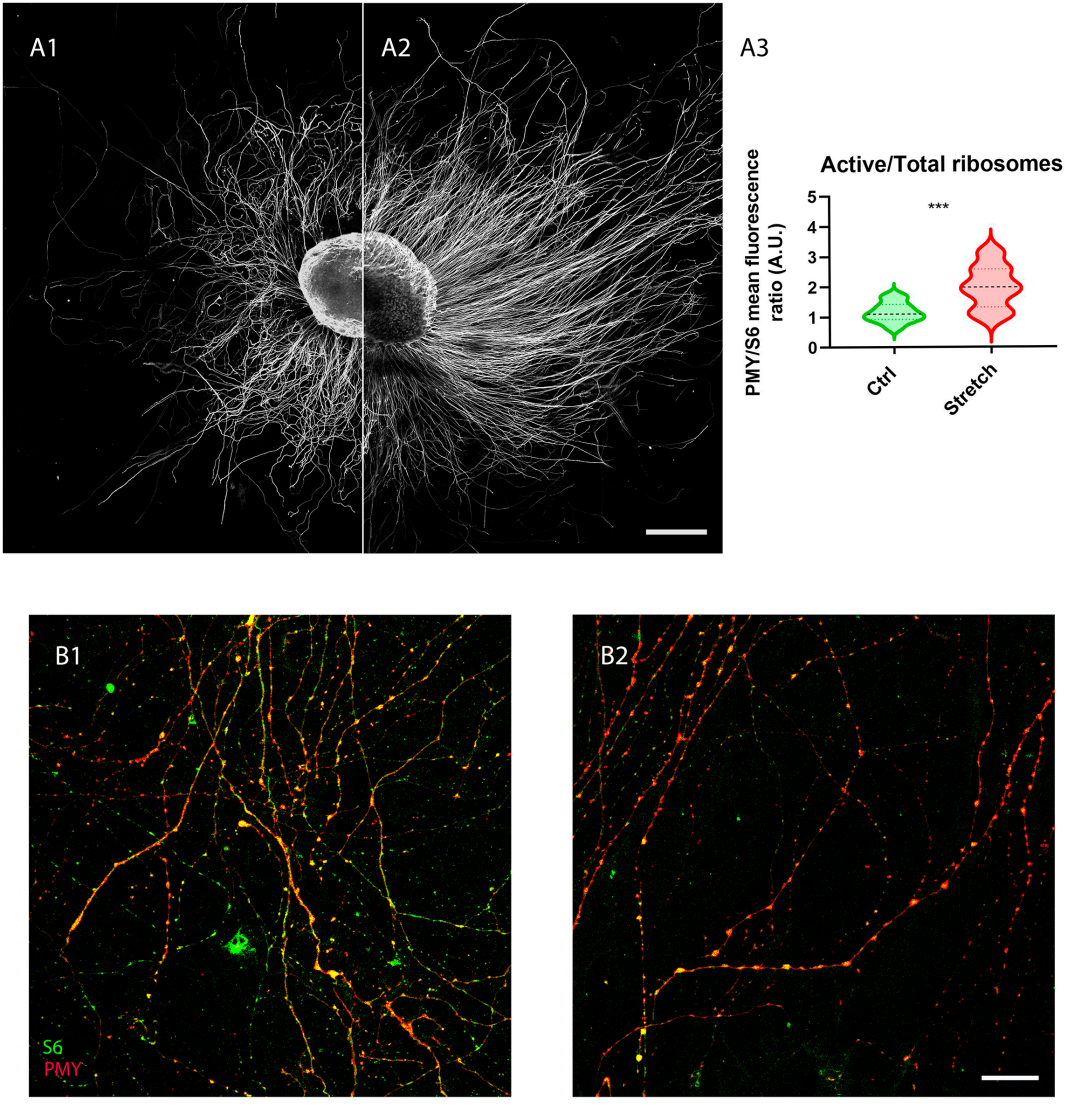
Nano-pulling stimulates the activation of local translation. **(A)** Unstretched **(A1)** and stretched **(A2)** DRG were cultured from DIV1 to DIV3 and the active ribosomes to the total were evaluated in the two conditions following the RPM method. **(B)** IF of active ribosomes and the total population (S6-positive) under spontaneous elongation **(B1)** and following nano-pulling **(B2)**. Puromycin (red) and S6 (green) staining. **(A3)** Analysis of the ratio between active ribosomes to the total in the two conditions. Violin plot (median and extremes as 1st and 3rd quartiles). *t* test for unpaired data. *p* = 0.0005. *N* > 12 DRG from 4 mice. Scale bars: **A** = 250 μm; **B**= 20 μm.

Given that local translation is one of the major mechanisms that sustain the addition of new mass in the axon, we analysed the fraction of ribosomes in active translation (PMY-positive) to the total in control samples S6-positive (Figure 3A1) and in those under nano-pulling (Figure 3A2). We observed a 72% increase (Figure 3A3; *p* = 0.0005) of the ratio of active ribosomes to the total in stretched samples (Figure B2) compared to those subjected to spontaneous regeneration (Figure 3B1).

### 3.3 Nano-pulling modifies microtubule

One mechanism responsible for the activation of local translation is the assembly of translational platforms, composed of late endosomes (LE), RNA granules and mitochondria (Cioni et al., 2019). Considering that the transport of translation machinery is MT-dependent (Broix et al., 2021), our previous studies suggested that the activation of translation could be related to an increase in MT stability (Falconieri et al., 2023b). We thus evaluated the ratio of acetylated to tyrosinated α-tubulin under the two experimental conditions. The rationale behind this is that acetylation is a tubulin post-translational modification which is associated with stability, whereas tyrosination is generally related to dynamic instability (Witte et al., 2008).

**Figure 4:**
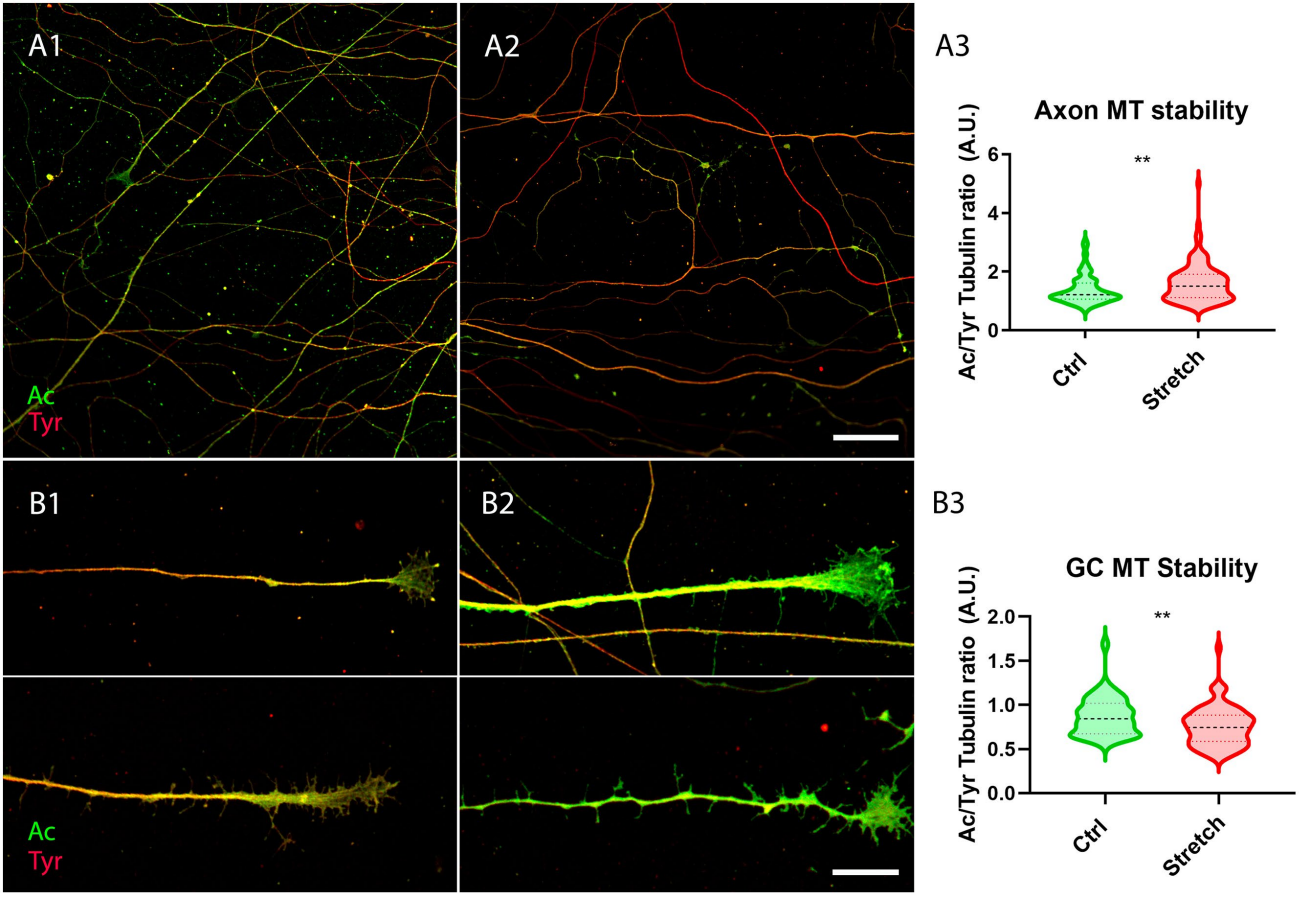
Microtubule stability increases in response to magnetic nano-pulling. **(A,B)** Immunostaining of acetylated (red) vs. tyrosinated (green) α-tubulin. **(A)** Evaluation of MT stability in control **(A1)** and stretched **(A2)** DRG axons. **(A3)** Quantification of the ratio between acetylated and tyrosinated α-tubulin. Violin plot (median and extremes as 1st and 3rd quartiles). Mann–Whitney test. *p* = 0.0013. *N* = 125 axons from 4 mice. **(B)** Evaluation of MT stability in unstretched **(B1)** and stretched **(B2)** DRG GCs. **(B3)** Quantification of the ratio between acetylated and tyrosinated α-tubulin. Violin plot (median and extremes as 1st and 3rd quartiles). *t* test for unpaired data. *p* = 0.0016. N > 75 GCs from 4 mice. Scale bars: **A** = 30 μm; **B** = 15 μm.

DRG were thus seeded on glass coverslips. They were treated with cell culture medium modified with MNPs after 4 h for subsequent nano-pulling (DIV0.17). The next day, DRG were randomly allocated to the control group (Ctrl) and the stretched group (Stretch). After 48 h of stimulation, we found an increase in the ratio of acetylated to tyrosinated α-tubulin in the shaft of axons subjected to magnetic nano-pulling (Figure 4A3; *p* = 0.0013). Conversely, regarding the growth cones (GC), we found a decrease in acetylated to tyrosinated α-tubulin of stretched samples compared to spontaneous elongation (Figure 4B3; *p* = 0.0016).

## 4 Discussion

In the present study, we validated the ability of nano-pulling to induce axon growth in an *in vitro* regeneration model that partially reproduces the complexity of a neural tissue, i.e., dissected DRG. Post-natal (but not adult) DRG explants are generally cultured in media modified with NGF for efficient stimulation of axon growth (Malin et al., 2007). Here we used NGF-free media to investigate if the mechanical stimulation is sufficient *per se* to promote a robust regeneration process. Previous studies have shown that nano-pulling is effective only if MNPs are within the axon where they can generate active mechanical force when exposed to a static magnetic field (De Vincentiis et al., 2020, 2023; Falconieri et al., 2023b). Compared to 2D cultures, whole ganglia present additional barriers to the penetration of MNPs, being characterised by similar features and complexity to *in vivo* neural tissues. MNPs may fail to reach their targets because of the presence of these barriers. First, they may not be able to pass the layers of connective tissue and glial envelope. Second, DRG contain non-neuronal cells with phagocytic activity (glial cells) which can internalise and quickly destroy the particles. Third, they may be poorly internalised by mature neurons. To validate the ability of MNPs to cross these barriers, soon after the attachment of the ganglia to the glass coverslip, DRG were grown in an MNP-modified medium for 3 days. The analysis of the ultrastructure confirmed that MNPs are able to penetrate the ganglia (Supplementary Figure S1) and to become localised inside neural neuronal cells (Supplementary Figure S2) and within the axon (Figure 1) but not in the nucleus, in line with previous studies carried out by our team on mouse hippocampal neurons (De Vincentiis et al., 2020). Many mono-dispersed nanoparticles were found in the axon, in proximity of the axolemma (green arrows) or freely dispersed within the axoplasm (red arrows), or in proximity of axonal compartment such as ER membrane, MT or mitochondria (cyan arrows). However, the data here collected are not sufficient to speculate about the mechanisms of internalization. Previous studies have shown that active coatings such as chitosan (Kunze et al., 2017) or wheat germ agglutinin (WGA) (Chowdary et al., 2019) or receptor-specific antibodies (Steketee et al., 2011), promote internalization by endocytosis, as proven by the accumulation of MNPs in intracellular vesicles. However, our MNPs – which present a glucuronic acid coating - did not show a tendency to cluster in endosome-like vesicles but, rather, they were found as isolated particles floating in the cytoplasm, attached to organelles or interacting with the cell membranes (Figure 1). Interestingly, we cannot exclude the axonal retrograde transport as it was reported that the uptake of negative charged nanoparticles by the axon of primary mouse cortical neurons and SH-SY5Y cells and their accumulation in the soma can occur by this mechanism (Lesniak et al., 2019). Direct penetration could be another alternative as it has been shown that some nanoparticles can be internalized directly (Zhang et al., 2011). More extensive studies are needed to elucidate the internalization mechanism. For the scope of the present study, it was crucial to confirm the presence of MNPs in DGR neurons together with the interaction between MNPs and one or more axonal components (e.g., membrane, ER and mitochondria), thus supporting the assumption that they could induce force generation when manipulated with magnetic fields. This was corroborated by our experimental finding which demonstrated that the nano-pulling increased the axon regeneration rate by about a 2-fold factor.

Additionally, we found that the ratio of acetylated versus tyrosinated α-tubulin decreased in the GC of stretched axons, highlighting the presence of dynamic MTs (Baas and Black, 1990) which is a typical feature of fast elongating axons. In fact, rapidly growing GCs are characterised by the presence of highly dynamic, exploratory MTs which translocate from the central domain to the transition domain of the GC during tip advance (Lee and Suter, 2008; Schaefer et al., 2008; Athamneh et al., 2017). Conversely, this ratio was found to increase in the shaft of stretched axons, highlighting the presence of more stable MTs (Takemura et al., 1992; Cappelletti et al., 2021). A correlation between the acetylation of MTs and their stabilization has always been observed in various studies on neurons (Morley et al., 2016; Yan et al., 2018; Teoh et al., 2022), as well as in other studies on non-neuronal models (Swiatlowska et al., 2020; Coleman et al., 2021). Consistently, in previous works we found that the force-induced stabilization of the MTs in the shaft causes their accumulation in stretched axons (De Vincentiis et al., 2020, 2023; Falconieri et al., 2023b). This mechanism seems to be independent on the technology used for force generation, as a similar trend was also observed with magnetic microposts, which are a different magnetically-activated technology that are capable of exerting higher forces extracellularly (Falconieri et al., 2022).

We previously demonstrated that the most direct consequence of the accumulation of MTs in the axon shaft is the positive modulation of the MT-dependent transport of vesicles and organelles (Falconieri et al., 2023b). Among organelles, mitochondria and endoplasmic reticulum are fundamental to sustain the production of proteins/lipids during regeneration. In energy production, a key role is played by mitochondria, which, at the cellular level, produce adenosine triphosphate (ATP), which when hydrolysed into diphosphate (ADP) releases the energy required for a variety of cellular functions. We have thus shown that nano-pulling results in an increase in the number of mitochondria compared to the control conditions (Figure 2C). This data on mitochondria in stretched ganglia are in line with previous findings on mice, human and chick isolated neurons (Lamoureux et al., 2010; De Vincentiis et al., 2023; Falconieri et al., 2023b). Endoplasmic reticulum is another key component for the production of proteins required for axon regeneration. Our experimental data revealed a strong increase in ER membranes in stretched samples compared to spontaneous regeneration (Figure 2B). The accumulation of ER membranes as an effect of nano-pulling has been observed in previous studies on isolated mouse hippocampal neurons (De Vincentiis et al., 2020; Falconieri et al., 2023b) and human neural stem cells (De Vincentiis et al., 2023). The generation of force with technologies other than nano-pulling has the same effect on ER and mitochondria accumulation (Falconieri et al., 2022). We also found an increase in synapsin I signal that is not surprising because synapsin I is a component of synaptic vesicle precursors that are transported on MTs along the axon (Guedes-Dias and Holzbaur, 2019). Interestingly, 48 h of stimulation were sufficient to detect a statistically significant increase in synapsin I signal. Accumulation of synaptic vesicles in response to force has been already demonstrated, such as for hippocampal neurons (Falconieri et al., 2023b) and Drosophila neurons (Siechen et al., 2009; Ahmed et al., 2012), regardless of the technology used to stretch neurons, i.e., MNPs (Falconieri et al., 2023b), stretchable polydimethylsiloxane (PDMS) substrates (Ahmed et al., 2012) or micropipette manipulation (Siechen et al., 2009).

Beyond ER and mitochondria, the third component required for the *in situ* production of proteins are ribosomes involved in local translation. Data obtained showed that nano-pulling resulted in a strong increase in active ribosomes in stretched axons (Figure 3). In our previous study, we observed that local translation also occurs through the formation of translation platforms between late endosomes and both active ribosomes and RNA granules (Falconieri et al., 2023b). The first group to theorize and demonstrate the formation of these platforms for local translation was Holt’s group in 2019. In their work they discussed the functional contacts between the components involved in the translation machinery for the production of newly synthesized proteins (Cioni et al., 2019). Mitochondria, along with late endosomes and RNA granules, serve as a key component of these platforms. The increase in the concentration of mitochondria, ER, and active ribosomes observed in the present work strongly supports the hypothesis of the formation of platforms used for local translation and protein production during nano-pulling.

In conclusion, we recently proposed a model according to which the mechanical stimulation induces (by an unknown mechanism) the stabilization of axonal MTs, resulting in the accumulation of MTs and MT-dependent transport of organelles and vesicles, which, in turn, favour the assembly of the “translational platform” and activation of local translation. This modulation of axonal transport and local translation is responsible for the mass addition required to sustain axon growth. The data collected in the present paper support the idea that the validity of this model, already proved for developing axons, could be extended to regenerating axons.

Indeed, we believe that the data presented in this work represent an encouraging starting point for exploiting nano-pulling to promote the regeneration of a neural tissue. The use of MNPs in biomedicine is widespread and the idea of using them to promote neuroregeneration is gaining great interest (Falconieri et al., 2019). This study on DRG shows that it is also possible to exploit nano-pulling at the tissue level. A limitation of this study is that, although DRG are more informative than 2D cultures, an organotypic model is not able to reproduce the complexity of an *in vivo* system. Another limitation is that, even if DRG project one branch in the PNS and another in the CNS, their neurons remain peripherals and have a high intrinsic capacity to regenerate in culture (Scott, 1977). Future studies will be needed to understand whether nano-pulling can also be used within a living organism by promoting regeneration in a damaged or diseased neural tissue.

The future perspective is to use the nano-pulling for the treatment of spinal cord injuries (SCI). MNPs have been already approved for clinical applications as diagnostic tools (Niemirowicz et al., 2012; Farinha et al., 2021). Magnetic stimulation in SCI requires uniaxial magnetic field gradients and moderate field strengths (<0.5 T). We believe that the knowledge collected here and in previous works (De Vincentiis et al., 2020, 2023) makes the nano-pulling a mature technology for pre-clinical validation in SCI rodent models for inducing the regeneration of damaged resident neurons or the differentiation of transplanted neural precursor cells in mature neurons and their integration with the host tissue.

## Data availability statement

The datasets presented in this study can be found in online repositories (https://zenodo.org/records/10071523).

## Ethics statement

The animal study was approved by Italian Ministry of Public Health and of the local Ethical Committee of University of Pisa. The study was conducted in accordance with the local legislation and institutional requirements.

## Author contributions

AF: Data curation, Formal analysis, Methodology, Writing – original draft, Writing – review & editing. PF: Formal analysis, Methodology, Writing – review & editing. LP: Formal analysis, Methodology, Writing – review & editing. VR: Conceptualization, Funding acquisition, Investigation, Project administration, Resources, Supervision, Writing – original draft, Writing – review & editing, Data curation, Visualization.
