# Supplementary figures and images for: Nano-pulling stimulates axon regeneration in dorsal root ganglia by inducing stabilization of axonal microtubules and activation of local translation
Source: Front Mol Neurosci. 2024 Apr 3;17:1340958. doi: 10.3389/fnmol.2024.1340958 (PMC11022966; doi:10.3389/fnmol.2024.1340958)

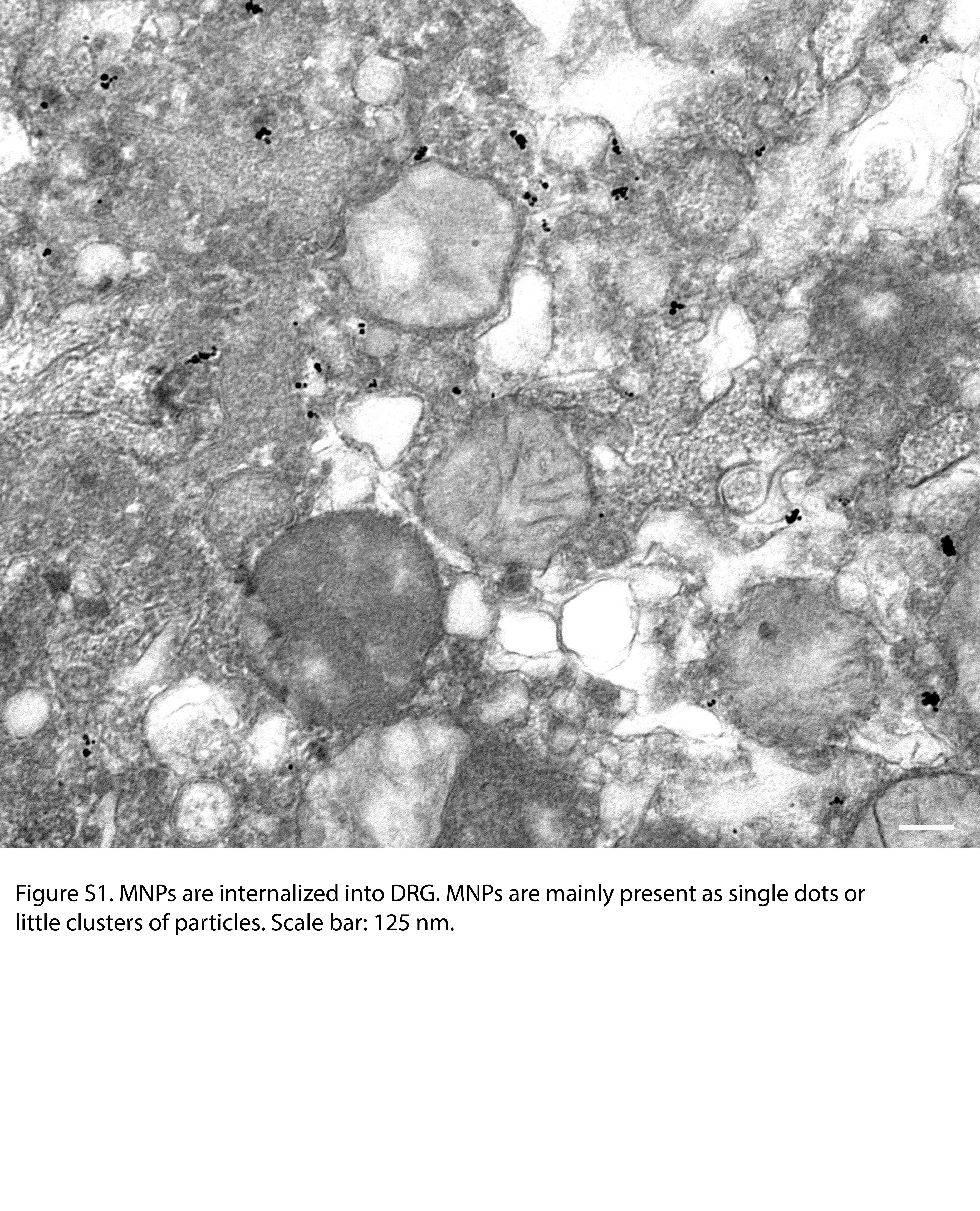

Supplement: Supplementary file 1 [file Image_1.jpg]

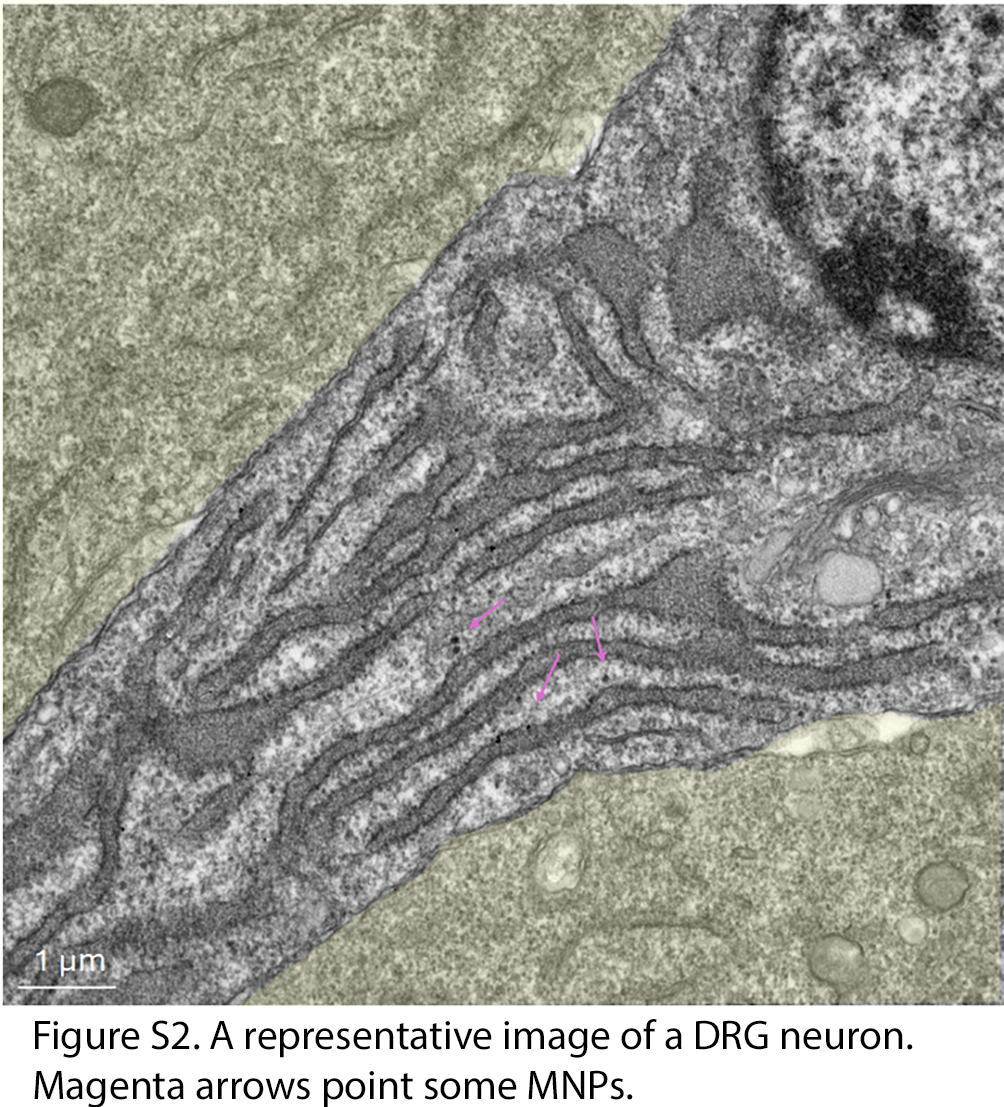

Supplement: Supplementary file 2 [file Image_2.tif]
